# Evaluation of remote digital postoperative wound monitoring in routine surgical practice

**DOI:** 10.1038/s41746-023-00824-9

**Published:** 2023-05-05

**Authors:** Kenneth A. McLean, Alessandro Sgrò, Leo R. Brown, Louis F. Buijs, Luke Daines, Mark A. Potter, Matt-Mouley Bouamrane, Ewen M. Harrison

**Affiliations:** 1 Department of Clinical Surgery, University of Edinburgh, 51 Little France Crescent, Edinburgh, EH16 4SA UK; 2 Centre for Medical Informatics, Usher Institute, University of Edinburgh, 9 Little France Rd, Edinburgh, EH16 4UX UK; 3 Colorectal Unit, Western General Hospital, Edinburgh, EH4 2XU UK

**Keywords:** Diagnosis, Gastrointestinal diseases, Infectious diseases

## Abstract

Remote digital postoperative wound monitoring provides an opportunity to strengthen postoperative community care and minimise the burden of surgical-site infection (SSI). This study aimed to pilot a remote digital postoperative wound monitoring service and evaluate the readiness for implementation in routine clinical practice. This was a single-arm pilot implementational study of remote digital postoperative wound monitoring across two tertiary care hospitals in the UK (IDEAL stage 2b, clinicaltrials.gov: NCT05069103). Adults undergoing abdominal surgery were recruited and received a smartphone-delivered wound assessment tool for 30-days postoperatively. Patients received 30-day postoperative follow-up, including the Telehealth Usability Questionnaire (TUQ). A thematic mixed-methods approach was used, according to the WHO framework for monitoring and evaluating digital health interventions. 200 patients were enroled, of whom 115 (57.5%) underwent emergency surgical procedures. Overall, the 30-day SSI rate was 16.5% (*n* = 33/200), with 72.7% (*n* = 24) diagnosed post-discharge. Usage of the intervention was 83.0% (*n* = 166/200), with subsequently 74.1% (*n* = 123/166) TUQ completion. There were no issues reported with feasibility of the technology, with the reliability (3.87, 95% CI: 3.73–4.00) and quality of the interface rated highly (4.18, 95%: 4.06–4.30). Patient acceptance was similarly high with regards to ease of use (4.51, 95% CI: 4.41–4.62), satisfaction (4.27, 95% CI: 4.13–4.41), and usefulness (4.07, 95% CI: 3.92–4.23). Despite the desire for more frequent and personalised interactions, the majority viewed the intervention as providing meaningful benefit over routine postoperative care. Remote digital postoperative wound monitoring successfully demonstrated readiness for implementation with regards to the technology, usability, and healthcare process improvement.

## Introduction

The use of telehealth within routine healthcare has become increasingly accepted practice^1,2^. There is now almost universal smartphone ownership among UK adults (93% in 2022^3^), vastly expanding the accessibility and feasibility of digital health interventions (DHIs). These interventions are viewed as an essential component to future delivery of surgical care^4^, including addressing the urgent surgical backlog in the post-pandemic recovery period^5^. However, there is widespread acknowledgement that the potential of DHIs have yet to be realised In healthcare systems^6^.

The early postoperative period is often associated with considerable patient morbidity, with surgical-site infection (SSI) being of particular concern following gastrointestinal surgery due to its high prevalence and burden posed to patients and healthcare systems^7^. There is increasing interest in the application of remote postoperative wound monitoring to both proactively diagnose SSI to facilitate potential perioperative interventions, as well as prevent over diagnosis and inappropriate antibiotic use. The “*Tracking wound infection with smartphone technology*” (TWIST) trial^8^ has been the only full-scale randomised control trial to be completed on this topic, demonstrating the feasibility, safety, and clinical efficacy of remote postoperative wound monitoring. However, the implementation of such complex health interventions within routine practice is challenging, often requiring substantial reorientation of healthcare services to integrate and sustain engagement from stakeholders^9^.

To date, few studies have evaluated the implementation of digital health interventions for postoperative wound monitoring in practice, with a lack of comprehensive evaluation of individual interventions^10^. Therefore, this study aimed to pilot a remote digital postoperative wound monitoring service and evaluate the readiness for implementation in routine clinical practice.

## Results

### Cohort characteristics

**Fig. 1:**
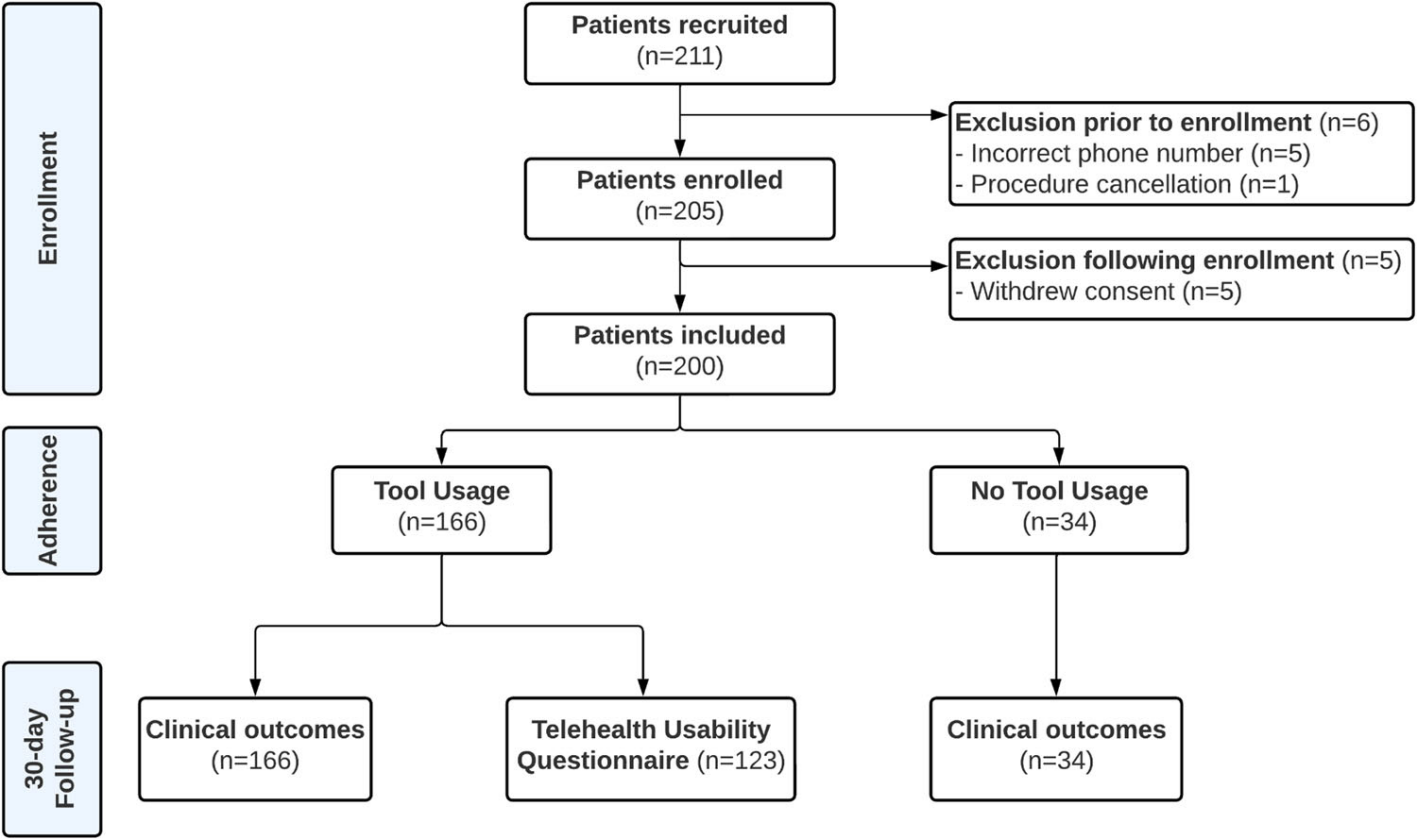
INROADE patient flow diagram. Depicts the patient flow for the INROADE study.

There were 211 patients recruited to participate in the INROADE study between the 1st July 2021 and 30th April 2022 (Fig. 1). There were 11 patients who did not participate, either from not receiving the intervention (incorrect phone number [*n* = 5] or procedure cancellation [*n* = 1]), or withdrew consent subsequently (*n* = 5). A total of 200 patients were included and eligible for follow-up.

The majority of patients included underwent emergency surgery (57.5%, *n* = 115/200), rather than elective operations (42.5%, *n* = 85/200) (Table 1). Operations were typically classed as major or complex major procedures (91.5%, *n* = 183/200), with the most common operations being appendicectomy (23.5%, *n* = 47/200) or cholecystectomy (23.5%, *n* = 47/200).

**Table 1:** Demographic and operative characteristics of study participants, overall and by usage.

		Remote postoperative wound surveillance			
		All (*n* = 200)	Usage (*n* = 167)	Non-usage (*n* = 33)	*p*
Age	Mean (SD)	48.0 (16.3)	48.4 (15.7)	46.4 (19.4)	0.508
Sex	Male	97 (48.5)	81 (48.8)	16 (47.1)	1.000
	Female	103 (51.5)	85 (51.2)	18 (52.9)	
Ethnicity	White	195 (97.5)	162 (97.6)	33 (97.1)	1.000
	BAME	5 (2.5)	4 (2.4)	1 (2.9)	
Socioeconomic deprivation	Quintile I-II	61 (30.5)	10 (29.4)	51 (30.7)	1.000
	Quintile III-V	139 (69.5)	24 (70.6)	115 (69.3)	
Body Mass Index (BMI)	Not obese	133 (66.5)	108 (65.1)	25 (73.5)	0.451
	Obese	67 (33.5)	58 (34.9)	9 (26.5)	
Immunosuppression	No	186 (93.0)	154 (92.8)	32 (94.1)	1.000
	Yes	14 (7.0)	12 (7.2)	2 (5.9)	
Diabetes Mellitus	No	187 (93.5)	156 (94.0)	31 (91.2)	0.825
	Yes	13 (6.5)	10 (6.0)	3 (8.8)	
Operative approach	Minimally-invasive	119 (59.5)	99 (59.6)	20 (58.8)	1.000
	Open	81 (40.5)	67 (40.4)	14 (41.2)	
Operative complexity	Minor/Intermediate	17 (8.5)	13 (7.8)	4 (11.8)	0.755
	Major	159 (79.5)	133 (80.1)	26 (76.5)	
	Complex Major	24 (12.0)	20 (12.0)	4 (11.8)	
Operative contamination	Clean-Contaminated	165 (82.5)	141 (84.9)	24 (70.6)	0.079
	Contaminated / Dirty	35 (17.5)	25 (15.1)	10 (29.4)	
Operative Urgency	Elective	85 (42.5)	73 (44.0)	12 (35.3)	0.458
	Emergency	115 (57.5)	93 (56.0)	22 (64.7)	

**Fig. 2:**
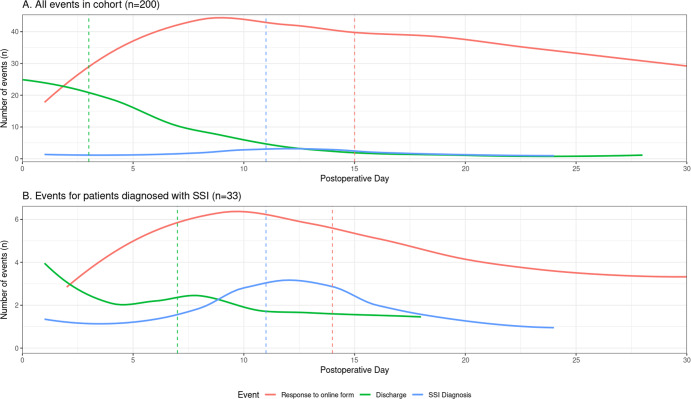
Patient postoperative events, including responses to the online form, discharge from hospital, and SSI diagnoses. Depicts the time-to-event (days) postoperatively for responses to the online form, discharge from hospital, and SSI diagnoses. This is shown for (**A**) All patients (**B**) Patients who were diagnosed with SSI within 30-days. Vertical lines represent median postoperative day of event.

Of the 200 patients included, overall usage of the tool was 83.0% (*n* = 166/200), with a median of 7.0 responses per active patient (IQR: 4.0 to 9.0, range: 1 to 20) (Fig. 2A). No significant differences were observed between those who did or did not use the tool (Table 1). Overall, 16.5%, *n* = 33/200 of the cohort developed SSIs in the 30-day postoperative period (Table 2). The majority were superficial (78.8%, *n* = 26/33) compared to deep (15.2%, *n* = 5/33) or organ-space SSIs (6.1%, *n* = 2/33), and classed as minor complications (Grade I-II: 93.9%, *n* = 31/33). The median time-to-diagnosis was 11.0 (IQR = 8.0–15.0) days, with 72.7% (*n* = 24/33) diagnosed post-discharge (Fig. 2B).

**Table 2:** 30-day Clinical Outcomes of study participants, overall and by usage.

		Remote postoperative wound surveillance			
*30-day clinical outcomes*		All (*n* = 200)	Usage (*n* = 166)	Non-usage (*n* = 34)	*p*
Community attendance	No	164 (82.0)	133 (80.1)	31 (91.2)	0.148
	Yes	36 (18.0)	33 (19.9)	3 (8.8)	
Hospital attendance	No	190 (95.0)	161 (97.0)	29 (85.3)	0.014
	Yes	10 (5.0)	5 (3.0)	5 (14.7)	
7-Day SSI rate	No	192 (96.0)	158 (95.2)	34 (100.0)	0.356
	Yes	8 (4.0)	8 (4.8)		
30-Day SSI rate	No	167 (83.5)	141 (84.9)	26 (76.5)	0.308
	Yes	33 (16.5)	25 (15.1)	8 (23.5)	
*30-day SSI-specific outcomes*		All (*n* = 33)	Usage (*n* = 25)	Non-usage (*n* = 8)	*p*
Time-to-Diagnosis (days)	Mean (SD)	11.3 (5.4)	10.0 (4.6)	15.4 (6.0)	0.012
Context of Diagnosis	Inpatient	9 (27.3)	7 (28.0)	2 (25.0)	1.000
	Post-discharge	24 (72.7)	18 (72.0)	6 (75.0)	
SSI Severity	Superficial	26 (78.8)	22 (88.0)	4 (50.0)	0.042
	Deep/Organ-space	7 (21.2)	3 (12.0)	4 (50.0)	
SSI-associated complication rate (Clavien-Dindo) ^a^	Grade I-II	31 (93.9)	24 (96.0)	7 (87.5)	0.432
	Grade III-V	2 (6.1)	1 (4.0)	1 (12.5)	

^a^No deaths associated with surgical-site infection were recorded within 30-days.

### Readiness for implementation

There was an 74.1% response rate (*n* = 123/166) to the technology usability questionnaire among those who used the intervention with no evidence of significant volunteer bias based on demographic or operative factors or 30-day SSI occurrence (Supplementary Table 3).

#### Technological Readiness

Among all participants, there were no issues with feasibility of the technology itself reported, although it should be noted that all patients recruited had to have a smartphone for participation. The quality of photographs supplied varied, with a minority featuring dressings or suboptimal angles. Nevertheless, 99.4% (*n* = 2134/2147) images received were perceived as sufficient quality to provide a degree of clinical insight. A minority (*n* = 4) required external help to take photographs due to the location of their wound (“*This is not a one size fits all and I required help from my family to take satisfactory, clear photos. It is less suitable for people who live alone or are elderly / infirm*”).

**Fig. 3:**
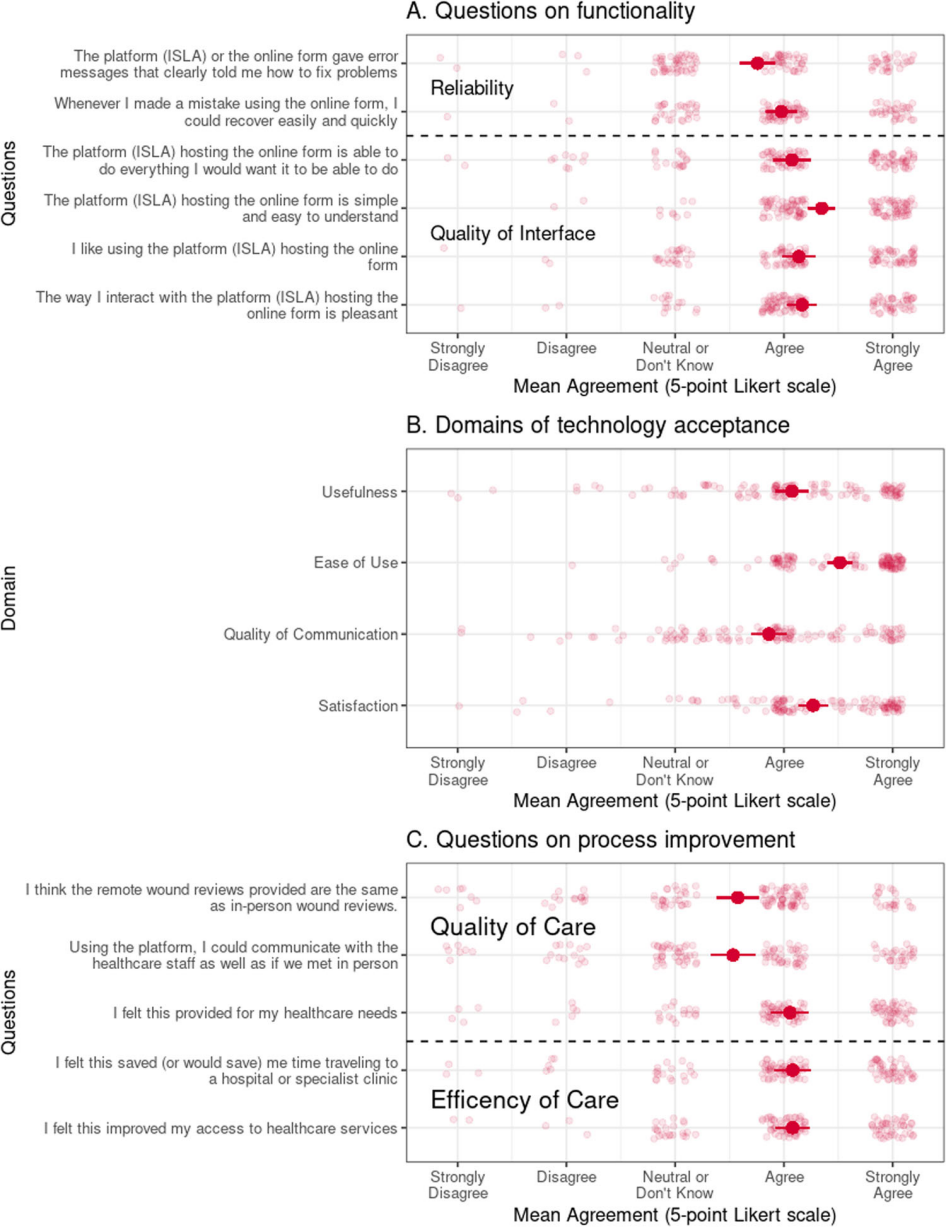
Patient rating of remote surgical wound assessment according to domains of the WHO Evaluation framework. Depicts the mean agreement (95% confidence interval) and individual responses (points) to the Telehealth Usability Questionnaire (TUQ). This is reported according the WHO Evaluation framework domains through: **A** Questions on functionality, grouped by domain; **B** Questions summarised by domain on patient acceptance; **C** Questions on process improvement, grouped by domain.

Overall, the functionality of the intervention was rated highly with regards to the overall reliability (mean = 3.87, 95% CI: 3.73–4.00) and quality of the interface (mean = 4.18, 95%: 4.06–4.30) (Fig. 3A). The lack of issues being most frequently commented upon (*n* = 5), with those reported focussing on difficulties uploading the image to the platform, with patients reporting challenges in uploading multiple photos in particular (“*I think it would be clearer if you could add more than one picture at the start, rather after answering the questions. That was confusing when using it first time around. I decided to just take one pic that covered all wounds for ease as someone else was taking the picture for me—it’s a faff to ask them to take the pic, then wait til I go through the rest and then get them to take more pics*.”). However, there were no significant differences observed according to subgroups (Supplementary Fig. 3).

Regarding improvements to the functionality of the specific tool, two sub-themes emerged. Firstly, six patients felt the scope of the tool should be widened to encompass further aspects of wound care or postoperative care beyond infection (“*It would be really handy if it wasn’t just about the wounds and also included advice on problems that have came after the surgery such as sickness etc as I had to contact the hospital as a result of this*.”). Secondly, two patients wanted to modify the schedule of reminders to complete based on the patient preference or the wound characteristics (“*As my wound was healing nicely it got a little bit tedious uploading photos*”).

#### Usability

Patient acceptance of remote postoperative monitoring was high across all domains assessed (Fig. 3B), with no significant differences observed according to subgroups (Supplementary Fig. 4). This was particularly evident for ease of use (mean = 4.51, 95% CI: 4.41–4.62), with this specifically highlighted as straightforward by seven patients, although others noted challenges (“*It only allowed me to upload 3 photos at a time, it would be easier if you could upload all photos each time*.”) or uncertainty about where free-text comments could be provided (“*It is confusing that the only place I can add a comment is when uploading the picture*”).

Similarly, questions related to satisfaction (mean = 4.27, 95% CI: 4.13–4.41) and perceived usefulness (mean = 4.07, 95% CI: 3.92–4.23) of remote postoperative monitoring were highly rated (Fig. 3B) with comments specifically highlighting improvement to their experience (“*It was really reassuring having this service, during a vulnerable time post op where it was easy to worry about the wound*.”) or empowerment in their own care (“*It gave me, as a first time patient and having never experienced this sort of thing before, confidence that the wound was healing and I was managing my wound well*.”). However, several patients noted reduced usefulness of remote monitoring when already receiving community follow-up care or existing experience with wound management (“*As a retired District nurse I felt I was on top of any wound care required*.”; “*Was constantly at my GP anyway*”).

Finally, whilst quality of communication was highly rated overall (mean = 3.86, 95% CI: 3.71–4.02), it was assessed less positively than other domains (Fig. 3B). There were no issues reported regarding the speed of response (“*Quick, easy way to have the wounds monitored*.”), and instead comments indicated this was principally driven by the desire for greater and more personalised interaction with the surgical team. For a minority of patients, the standardised responses received were be perceived as automated (“*The lack of personalisation in the response made it feel as though it was not being reviewed by a human … I was confident in the system but the lack of personal interaction may force others to seek further help if questions arose*.”), with patients desiring a greater degree of human contact through 2-way messaging to facilitate communication (“*I could communicate through the tool however the SMS I received back could not be replied to, so there isn’t really a conversation*.”).

#### Process improvement

There were no issues reported with feasibility of the technology, with the. Patient acceptance was similarly high with regards to ease of use, satisfaction, and usefulness. Quality of communication was rated lowest, related to the desire for more frequent and personalised interactions with the surgical team. Nevertheless, the majority viewed the intervention as providing meaningful benefit over routine postoperative care, in terms of the quality and efficiency of care.

Patients receiving remote digital postoperative wound monitoring generally perceived an improvement to the efficiency of postoperative care experienced (mean = 4.08, 95% CI: 3.93–4.23) (Fig. 3C), particularly the convenience and improved access to care (“*My wound was being monitored … in the convenience of my own home … I think this has been a great service and helped me to seek further help from GP services*.”). This was also reflected in views of the quality of care received (mean = 3.72, 95% CI: 3.56–3.89), with most agreeing it addressed their postoperative healthcare needs regarding their wounds (Fig. 3C). Several comments (*n* = 6) specifically indicated there was a meaningful benefit over routine postoperative care (“*As I wouldn’t have received in person wound reviews so difficult to compare, in my opinion online reviews are way better than no reviews*”). Nevertheless, responses were less positive regarding the equivalence to in-person assessment (Fig. 3C), with five comments highlighting this (“*It would be wonderful if we had district nurses who could pop in and check on your wound in person but that’s not going to happen so I was delighted to have the online tool*”) or their preference for in-person assessment (“*It is not a substitute for proper health care and human contact particularly for older people who perhaps live alone and are anxious about their care or procedure, or other aspects of healthcare other than wound management … A photo and a text is a step in the right direction but not to replace face to face evaluation*”). Overall, there were no significant differences observed according to subgroups (Supplementary Fig. 5).

## Discussion

This implementation study provided sequential assessment of remote digital wound monitoring^8^, and demonstrates high levels of patient engagement and acceptance within an undifferentiated general surgical population. The majority of respondents viewed the intervention as providing a meaningful benefit over routine postoperative care with regards to their postoperative experience and the quality and efficiency of care received. Furthermore, there was no evidence of significant differences in accessibility of the intervention or adherence among patient subgroups investigated. This demonstrates the intervention continues to be feasible and an effective adjuvant to enhance postoperative care.

Prior to implementation of any novel digital health intervention in routine care, the intervention itself and the technology used to deliver it must be sufficient to fulfil the clinical need. This intervention is composed of patient-generated data involving wound-based images and patient-reported outcomes. While these data are viewed as an increasingly important tool in healthcare to improve care and reduce healthcare inequities, there are ongoing concerns regarding the clinical relevance, quality, quantity, and interpretability of data received for clinical decision-making^11,12^. Previous studies of remote wound monitoring have highlighted the issue of variable quality of patient-generated images^13–15^. Nonetheless, these are typically reported to be of sufficient quality for clinical assessment, including in INROADE. This may be able to be further minimised through additional patient-orientated guidance^16^ or support tools such as flagging of common issues such as image blurring^16^, combined with direct provider-patient feedback to retake if an image is unsuitable. However, particularly for those with physical impairments and limited home support, the challenge of self-imaging abdominal wounds may perpetuate barriers to accessing healthcare that are already experienced in these patient groups. Nonetheless, there was no evidence of significant differences in accessibility of the intervention or adherence among patient subgroups investigated. Furthermore, few issues were noted with the feasibility or core functionality of the platform used to host the intervention, with INROADE benefitting from deployment on a platform already in routine use within the NHS.

The intervention must also demonstrate clear benefit to both patients and healthcare services. The majority of patients were satisfied with the intervention, felt it provided for their healthcare needs, and that it had a meaningful benefit over routine postoperative care (Fig. 3). This is consistent with previous studies on remote postoperative wound monitoring, which report similarly high patient satisfaction, perceived usefulness^13,17–19^, improved quality^8,15,18^ and efficacy of care^8,18^. However, patients were less likely to view this as a direct replacement for in-person care (Fig. 3C). This may be related to the trend of moving towards scheduled postoperative follow-up for only high-risk patients, limiting opportunities for healthcare interaction if not actively sought and leading to perceived lack of support in the community^20,21^. This supports the approach adopted with our intervention which was designed as a triage tool to enhance existing healthcare pathways, rather than to replace in-person assessment (particularly as diagnosis of SSI requires clinical examination^22^). However, a key factor which differentiated the intervention from in-person or telemedicine consultations was the lack of direct, two-way patient-clinician communication^23^. While not an aspect of our existing intervention, the integration of telemedicine consultation for select patients to inform the triage decision may further enhance acceptance and accuracy of the assessment process. Furthermore, a minority of patients specifically suggested the scope of the tool should be widened to encompass further aspects of wound care or postoperative care. While the focus of this intervention was on surgical wounds, there is increasing interest in broader perioperative monitoring in the literature^24^, particularly how these can be used within “virtual ward” pathways to prevent admission and facilitate discharge^25^. While this may further reduce health service utilisation and present an avenue for future development, these potential benefits must be balanced with the burden on patients to complete a more complex series of patient-reported outcomes^26^, and on health services to review and respond^27^.

INROADE is a pragmatic interventional study providing comprehensive evaluation of remote digital postoperative wound monitoring and expanding the evidence following the original trial^8^. Previous work has demonstrated clinical efficacy of this intervention in accurately triaging emergency surgery patients according to their risk of SSI, reducing healthcare utilisation, and allowing SSI to be diagnosed earlier in the postoperative period^8^. Instead, INROADE adopted an implementational approach and included an undifferentiated population of general surgical patients to reflect those treated in routine practice, with high levels of engagement and acceptance. This was evaluated using a validated tool (TUQ)^28^, which unlike other interventions^14,15,17–19,29–32^, encompassed the majority of key domains highlighted by the WHO framework for monitoring and evaluating DHIs^33^. This involved specific investigation of accessibility among key patient demographic groups regarding overall adherence (Supplementary Table 3) and within individual domains (Supplementary Figs. 3–5) which has not been previously performed for DHIs for remote postoperative monitoring^34^. This evaluation also builds upon previous evidence of clinical efficacy and process improvement demonstrated in the original TWIST trial to support recommendations regarding the readiness for implementation^8^. Finally, significant barriers to implementation of digital health interventions within routine care have been addressed in order to facilitate wider adoption. In its current iteration, the tool is deployed on an established online platform already in routine use and does not serve an independent diagnostic function. Therefore, the intervention described is not classified as a medical device and so does not require there for additional regulatory approvals prior to routine clinical within the UK^35^.

This implementation study also had several limitations. Firstly, the generalisability of these results should be considered. INROADE was conducted across two centres in a single health board in a high-income country. As such, the results may be affected by local healthcare policies, and patients may have greater smartphone-ownership and internet access than other locations^36^. As such, further independent evaluation at a larger scale would provide additional evidence of effectiveness and generalisability of this intervention to other healthcare contexts. Furthermore, INROADE was conducted during the COVID-19 pandemic. Although recruitment did not occur during periods of high community prevalence, patients may still have faced additional challenges or reluctance to access care post-discharge. This may have increased the acceptance and perceived utility of remote digital postoperative wound monitoring without the influence of the COVID-19 pandemic. Further work would be required to confirm this persists beyond the pandemic. Secondly, the results presented may not be representative of formal adoption of remote digital postoperative wound monitoring into the local care pathway. Individual patient consent was required in order to enrol patients into the study, risking the introduction of volunteer bias into the patient cohort recruited. As such, higher patient engagement may have been observed in comparison to enrolment within routine practice. This may have been compounded by the requirement for smartphone ownership to be enrolled, which risks the “digital divide” perpetuating potential inequities in care, particularly for different ethnic backgrounds or older patients^37^. While there has been no differences in adherence by age identified in INROADE, there were few patients at the extremes of age included and previous TWIST trial demonstrated patients who were excluded on the basis of having no smartphone access were significantly older^8^. While the ongoing expansion in smartphone ownership on a global basis ensures this will have diminishing significance over time^3^, all efforts should be made to address the impact of this inequity. At a service level, traditional methods of postoperative communication such as letter or telephone contact will need to continue^38^. Furthermore, digital services will need to be adaptable to individual digital literacy and physical limitations with patients identified prior to discharge as either: (1) independent with minimal digital literacy training needs; (2) potentially independent with digital literacy training; (3) requiring support to participate, whether from a connected person or community healthcare services. Thirdly, evidence for this intervention to date is predominantly from the perspective of patients or the health system rather than healthcare staff. Nevertheless, this intervention had input from surgical and family medical practitioners throughout the development process to ensure the online form reflects their clinical assessment of surgical wounds. Finally, despite high adherence and response rates among those who used the tool, there is a risk of results being influenced by volunteer bias. There were no significant differences observed between these groups (Table 1, Supplementary Table 3), although the study was not powered for this endpoint and so these represent overall small subgroups. Furthermore, there has been no formal validation of the online form, and so particularly inter-rater reliability and cross-cultural validity should be considered prior to implementation in other contexts. Future work may identify specific populations which may benefit from adaptations to further improve engagement or to reduce potential barriers to access.

As the only intervention of remote digital postoperative wound monitoring supported by high-quality trial evidence, future work should focus implementation in routine surgical care^39^. This has been facilitated through adoption of the intervention as a flagship case study of the NHS Transformation Directorate “*Perioperative digital playbook*”—a resource used internationally to provide evidence and examples of best practice to clinical teams and organisations to integrate digital tools into healthcare services^40^. Therefore, in addition to shaping future research directions on the topic, this work will directly influence future surgical care pathways. However, successful implementation of these complex interventions is dependent not just on the readiness of the intervention itself, but the context and process in which it is delivered^41,42^. Sustainable integration of remote monitoring into existing care pathways will require restructuring of local health services and redistribution of staff to deliver. Ineffective “normalisation” of these complex interventions within local healthcare environments is a key factor in failure of implementation in routine practice, and therefore further work is required to understand local and systemic barriers and facilitators^41^. Moreover, the burden to healthcare staff and so the cost-effectiveness of the intervention will depend on the volume and frequency of responses to review, balanced with the resultant reduction to health service utilisation^8^. While automated score- or algorithm-based assessment of submissions may reduce this burden to deliver^43^, this would require additional regulatory approval^35^ alongside proactive steps to address both patient apprehensions^44^ and potential bias in models perpetuating healthcare inequities^45^.

Remote digital postoperative wound monitoring successfully demonstrated readiness for implementation with regards to technological readiness, usability, and process improvement. This builds upon recent randomised control trial evidence comparing to routine clinical care, and early-stage work from independent research teams to support the value and appropriateness of this form of postoperative follow-up^34^. The majority of respondents viewed the intervention as providing a meaningful benefit over routine postoperative care. However, this was generally not considered as equivalent to in-person assessment, in particular regarding the lack of direct, two-way patient-clinician communication. This validates the approach adopted by the intervention here: a triage tool to enhance existing healthcare pathways, rather than to replace in-person assessment. With the increased demand on surgical health services during post-pandemic recovery, novel tools are needed to ensure stretched healthcare resources can be appropriately allocated to patients requiring clinical intervention, as well as enhancing community support for all patients in the postoperative period.

## Methods

### Study design

This is a multi-centre mixed-methods evaluation of a single-arm pilot implementational study of a digital health intervention for remote postoperative wound monitoring. This is a sequential evaluation of the intervention developed in the “*Tracking wound infection with smartphone technology*” (TWIST) trial^8^—a previous randomised control trial of the effectiveness of this intervention over routine postoperative care. It is reported according to mERA (mobile health evidence reporting and assessment) guidelines^46^ and IDEAL (Idea, Development, Exploration, Assessment, Long-term study) guidelines for stage 2b studies^47^.

The “*ImplementatioN of Remote Surgical wOund Assessment During the coviD-19 pandEmic*” (INROADE) study was conducted across two tertiary care hospitals in a large health board in the United Kingdom, serving an urban-rural population of over 800,000. It was reviewed and approved by West of Scotland Research Ethics Committee (21/WS/0046) and pre-registered on ClinicalTrials.gov (NCT05069103). Adult inpatients (aged ≥ 16 years) who were consented to undergo abdominal general surgery (at least one surgical incision into the peritoneal cavity or gastrointestinal tract) were screened for eligibility. Key inclusion criteria were smartphone ownership (with internet access) and the capacity to provide informed consent. Patients were excluded based on self-reported visual impairment which would prevent interaction with online resources. Written informed consent for each patient was obtained in line with Good Clinical Practice (GCP) standards. Sample size and power were explored over a range of plausible effect sizes (Supplementary Fig. 1). With 104 patients effect sizes of patient agreement in the region of 0.33 were able to be examined at 90% power. Based on previous work^8^, a usage rate for the intervention of 65% and a response rate to questionnaires of 80% was anticipated, and so a target sample size of 200 patients was required.

### Intervention

Digital health interventions like remote postoperative wound monitoring represent “complex health interventions”^48^. Patients engage in closed loop communication with healthcare staff using the technology (involving timely review and clinical recommendations based on information supplied), and then with the healthcare system if further clinical input is recommended (Supplementary Fig. 2). Enrolled patients had a personal hyperlink to the online form automatically sent by short-messaging system (SMS) to their smartphones. Patients could complete the online form (tool) throughout the early postoperative period (postoperative day 1–30), This included an image of their surgical wound(s), and a series of patient-reported outcomes (PROMs) related to signs and symptoms of SSI (Supplementary Table 1). Patients could provide additional context using free-text. This is consistent with this aspect of the intervention previously described in the original TWIST trial^8^, however there were three key modifications:

1. The tool was hosted on an industry-developed online platform (ISLA Care Ltd) already in routine use in NHS care pathways, instead of a research-orientated database (REDCap). ISLA Care Ltd acted as the data processor on behalf of the study sponsors, with individual patient consent and NHS information governance approval.
2. There was an increased frequency of routine requests to complete the tool over the 30-day period (every 3 days, compared to requests on days 3, 7, and 15 in the TWIST trial^8^).
3. There were branching questions added to quantify changes over time of each symptom (whether “*new onset*”, or “*worse*”, “*same*”, or “*better*” compared to the last submission).

Submission of a response by a patient generated an automated alert for review of the information by qualified clinician trained to recognise SSI. The evidence of SSI on patient-reported symptoms and wound images was classified as either: (1) no clear evidence of SSI present (low-risk), but with recommendation to attend healthcare services or submit a further form if ongoing concerns; (2) possible evidence of SSI (medium-risk), with recommendation to attend community healthcare services for clinical review; or (3) probable evidence of SSI (high-risk), with recommendation to attend emergency services at their treatment centre for clinical review. Submission of this clinical recommendation was performed within a target of 24 h from the time of first alert, with this response communicated to the patient through SMS on an automated basis on submission by the reviewing clinician (Supplementary Fig. 2).

### Data collection and 30-day follow-up

Further sociodemographic and operative data were collected based on clinically relevant risk factors for SSI or potentially disadvantaged populations. These included age, sex, ethnicity (White, or Black, Asian and other minority ethnic groups [BAME]), obesity (body mass index [BMI] ≥ 30 kg/m^2^), social deprivation (corresponding to the index of multiple deprivation (IMD) quintile^49^), diabetes mellitus, immunosuppression (known HIV positive status, corticosteroids, chemotherapy received within 6 weeks, or other immunomodulating drugs), operative approach (open or laparoscopic), operative complexity (minor/intermediate or major / complex major according to the BUPA Schedule of Procedures^50^), and CDC surgical wound classification^22^ (Clean / Clean-Contaminated, or Contaminated / Dirty).

All patients received 30-day postoperative follow-up following a standardised format. Patients who utilised the intervention were asked to complete the Telehealth Usability Questionnaire (TUQ)^28^ to evaluate their experience and opinions regarding the intervention. This could be completed online or via telephone, and involved 24 questions encompassing 6 domains: usefulness, ease of use, quality of interface, quality of communication, reliability, and satisfaction. 30-day clinical outcomes were assessed using a combination of: (1) the validated Bluebelle Wound Healing Questionnaire^51^; (2) electronic patient record review; and (3) review of wound logs which documented any wound reviews in the community (returnable in a pre-paid envelope). On the basis of these three sources of information, clinical researchers (trained in applying the CDC criteria^22^) determined if SSI was present. Further outcomes considered healthcare attendance for wound review, and Clavien-Dindo grade of SSI-associated complications^52^ (divided into “*minor*” [Grade I-II] and “*major*” [Grade III-V]).

### Data analysis

A mixed-methods approach was used^53^, and all data were pseudo-anonymised prior to analysis. The results were synthesised according to the WHO framework for monitoring and evaluating digital health interventions^33^. This framework encompasses several domains regarding: (1) Technological readiness: which encompassed both functionality (whether the technology fulfils the intended purpose) and feasibility (whether the technology is deliverable in the study context); (2) Usability: the quality of the interaction between the user and the technology, in terms of the adherence, acceptance and accessibility among patients or staff; (3) Healthcare impact: which encompasses clinical efficacy (how the technology influenced patient outcomes) and process improvement (how the technology improves service delivery, in terms of the cost, efficacy, quality or utilisation of healthcare). However, the state of maturity (including prior evidence) regarding DHIs guide which types of evaluation are most appropriate and so not all are expected to be evaluated within a single study. Patient adherence was assessed according to the number of responses submitted within 48 h following a routine request. Non-adherence was defined as no usage of the tool.

For quantitative data, statistical significance was set a priori at *p* < 0.05 and analysis was performed using R 4.1.1 (R Foundation for Statistical Computing, Vienna, Austria), with packages including *tidyverse* and *finalfit*. Numerical data were summarized as mean (standard deviation) or median (interquartile range) based on visual and statistical evaluation for normality, with appropriate tests for parametric or non-parametric data performed. However, in line with accepted practice regarding Likert scales^54^, the degree of respondent agreement with statements were analysed as continuous data ranging from 1 (“*strongly disagree”*) to 5 (“*strongly agree”*) using Welsh’s *t*-test. Categorical data were cross-tabulated, and differences tested using χ^2^ or Fisher’s exact tests. The TUQ items were mapped to the WHO evaluation framework (Supplementary Table 2), but continued to be summarised according to the intended domains as an overall mean score where appropriate. A priori patient subgroups were explored to investigate accessibility, including: age (<65 years, or ≥65 years old), social deprivation (IMD quintile 1–2, or IMD quintile 3–5), and operative urgency (elective surgery or emergency surgery). Patients with missing data were excluded on an analysis-by-analysis basis.

Qualitative data based on free‐text responses were visually inspected and any text which might identify an individual was anonymised prior to analysis. Every effort was made to retain the semantic integrity of the text, and any such amendments were indicated in direct quotations. A thematic analytic approach was adopted, and the domains of the WHO framework for monitoring and evaluating digital health interventions^33^ provided initial themes. Transcripts were initially read by one author (KAM), and a coding frame was devised to identify further subthemes^55^. These hierarchical codes generated were discussed with the wider team and, where appropriate, these themes and subthemes identified were triangulated with quantitative data collected^56^.

### Reporting summary

Further information on research design is available in the Nature Research Reporting Summary linked to this article.

## Supplementary information

Supplementary Material

Reporting Summary

## Data Availability

The datasets generated during and/or analysed during the current study are available from the corresponding author on reasonable request.

## Code availability

The code generated and used during the current study are available from the corresponding author on reasonable request.
