# Supplementary material for: Evaluation of remote digital postoperative wound monitoring in routine surgical practice
Source: NPJ Digit Med. 2023 May 5;6:85. doi: 10.1038/s41746-023-00824-9 (PMC10161985; doi:10.1038/s41746-023-00824-9)
Supplement: Supplementary file 1 — Supplementary Material [file 41746_2023_824_MOESM1_ESM.pdf]

Supplement

Supplementary Figure 1: Statistical Power Curve

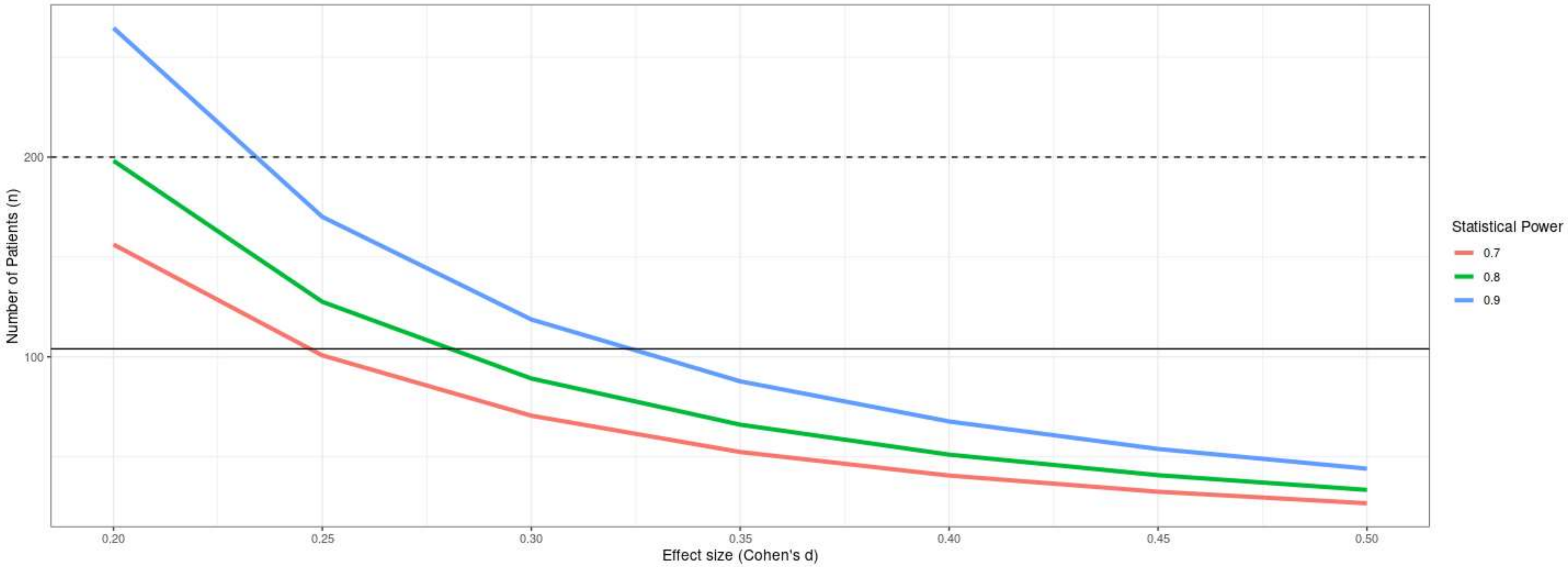

Supplementary Figure 2: Intervention Schemata

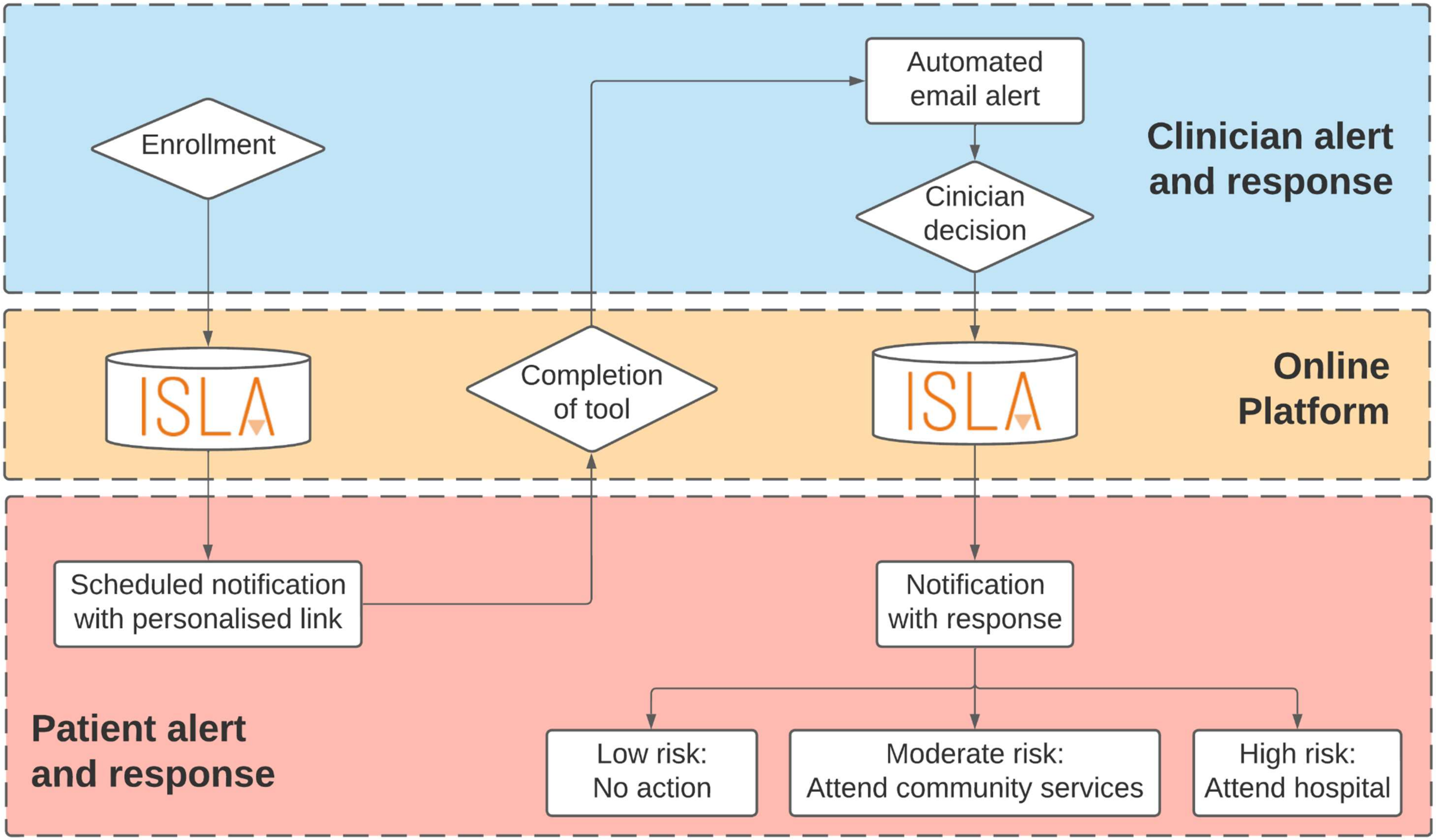

Supplementary Figure 3: Patient rating of functionality of the TWIST intervention, by subgroup

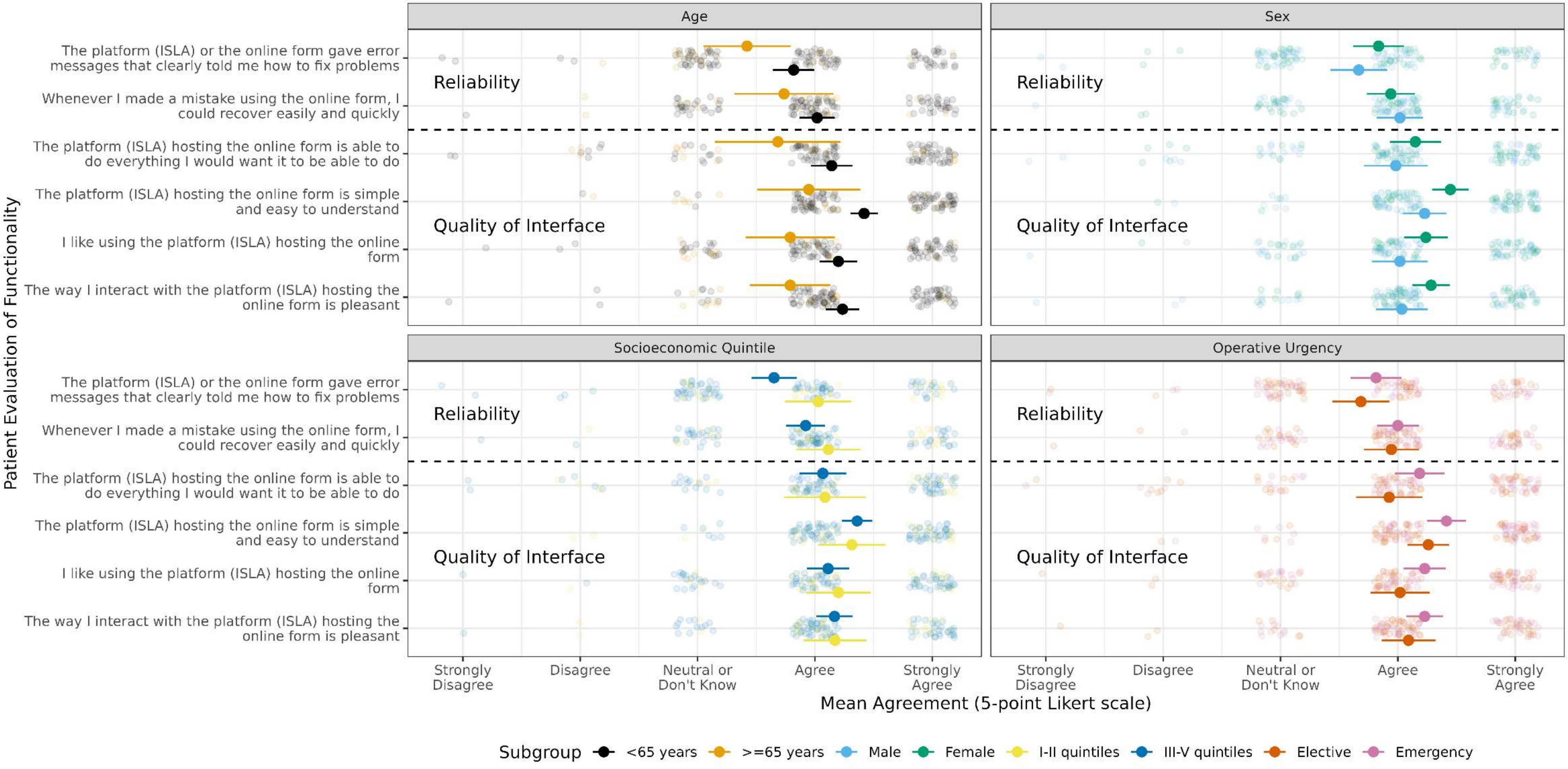

Supplementary Figure 4: Patient rating of technology acceptance of the TWIST intervention, by subgroup

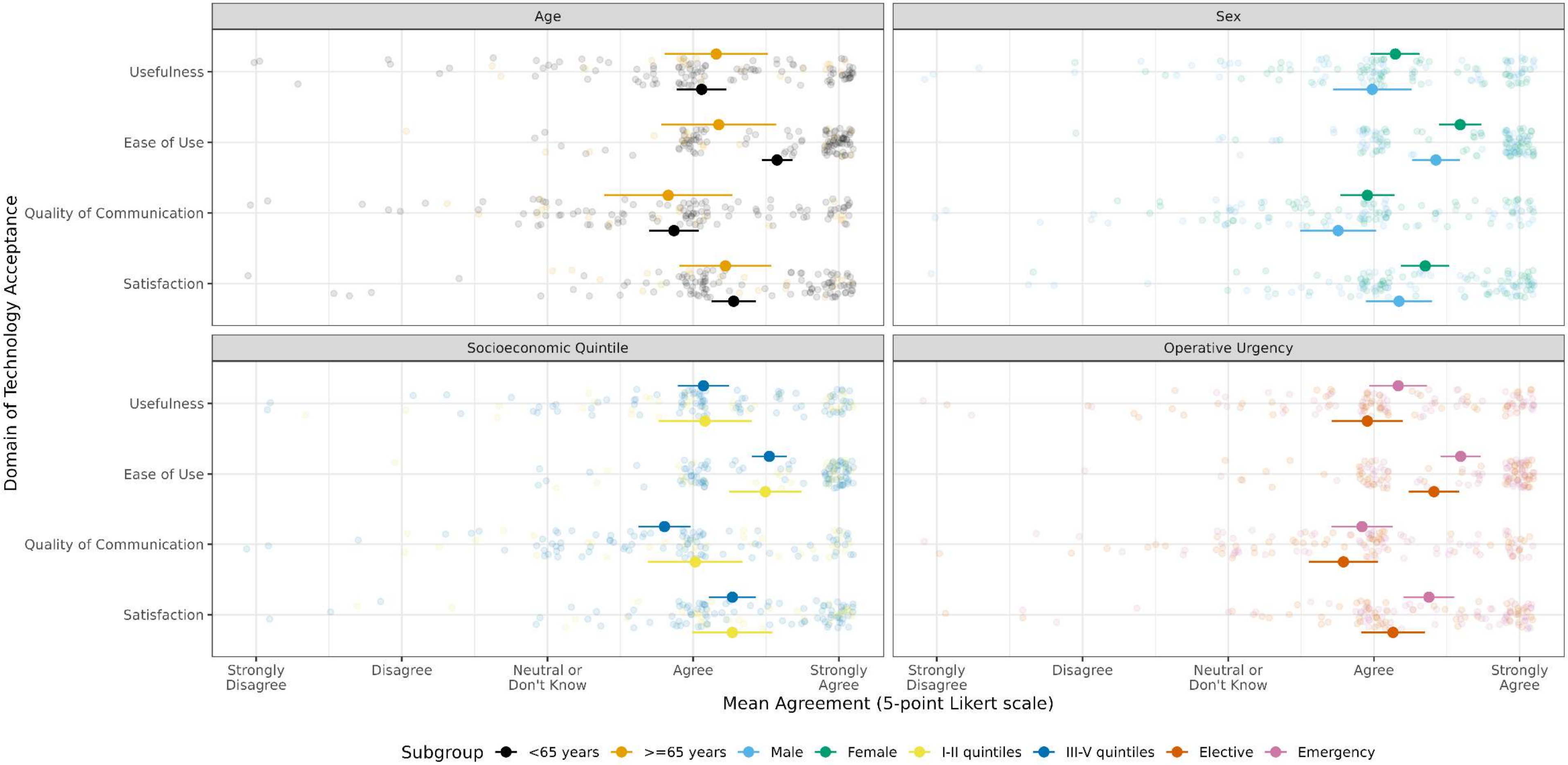

Supplementary Figure 5: Patient rating of process improvement of the TWIST intervention, by subgroup

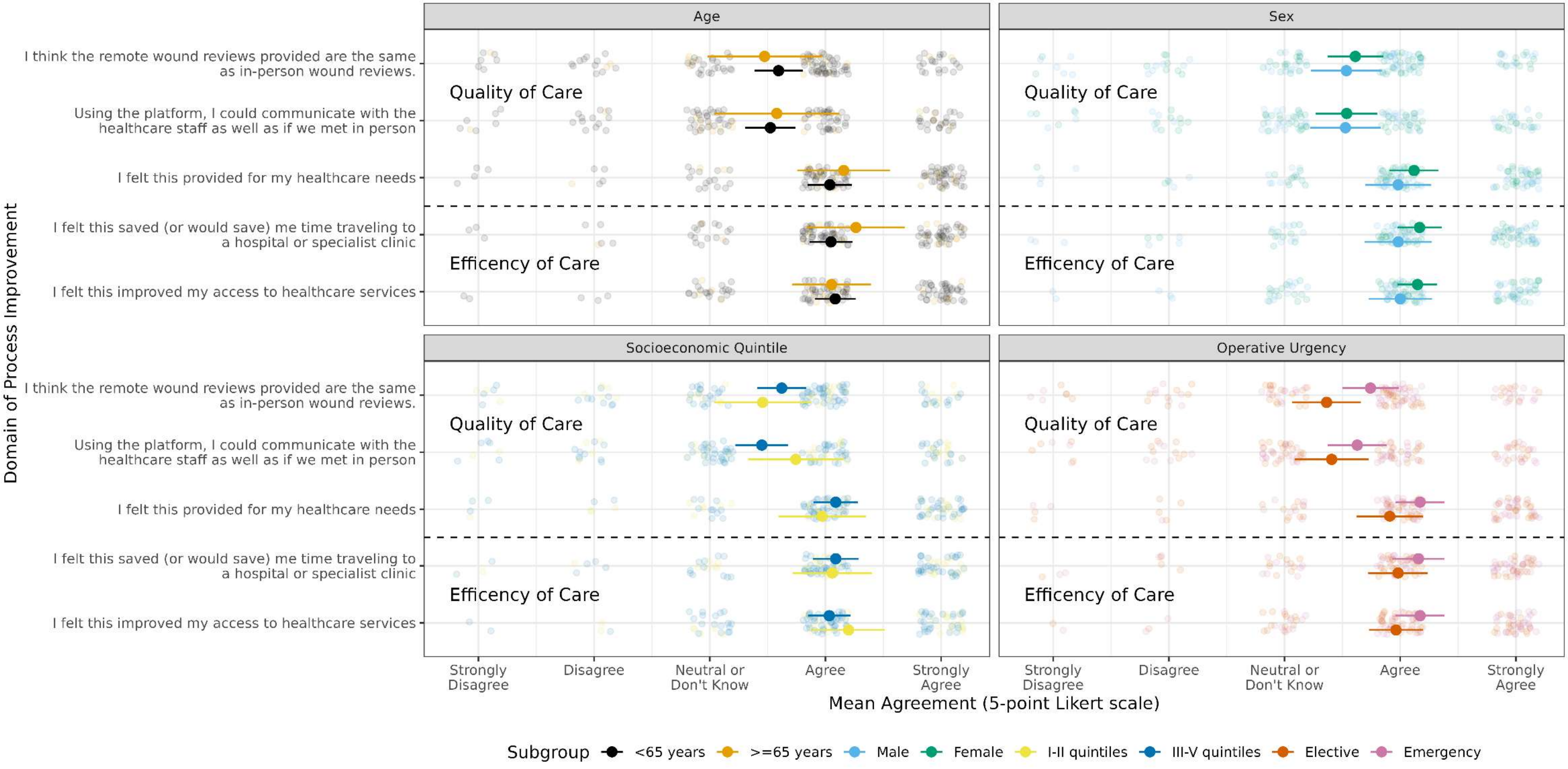

Supplementary Table 1: Smartphone-delivered wound assessment tool

Stem question	Options	Branching questions (if "Yes" selected)	Options
1. Has the wound been painful to touch	No, Yes	a) Over the past day, do you feel the pain is:	Worse, Same, Better, New (first time noticed)
2. Is there liquid coming from the wound site	No, Yes	a) Over the past day, has the amount of fluid leaking:	Increased, Stayed the same, Reduced, New (first time noticed)
		b) What is the colour/consistency of the fluid at present?	Clear, Bloody, Yellowish, Thick/yellow, Green/brown
3. Is there redness spreading away from the wound?	No, Yes	a) Over the past day, has the redness become:	Worse (redder or bigger), Same, Better (less red or smaller), New (first time noticed)
4. Has the area around the wound become swollen?	No, Yes	a) Over the past day, has the swelling become:	Worse (more swollen or bigger area), Same, Better (less swollen or smaller area), New (first time noticed)
5. Was the area around the wound warmer than the surrounding skin?	No, Yes	a) Over the past day, has the warmness become:	Worse (hotter to touch), Same, Better (less hot to touch), New (first time noticed)
6. Have the edges of any part of the wound separated/gaped open of their own accord?	No, Yes	a) Over the past day, has the opening wound become:	Worse (more opening up), Same, Better (closing up), New (first time noticed)
		b) Has the deeper tissue separated?	Just the skin, Deeper
7. Have you had, or felt like you have had, a raised temperature or fever? (fever > 38 °C)	No, Yes	-	-
8. Have you been diagnosed with a wound infection since surgery?	No, Yes	-	-
9. Please upload a photograph of your wound.	-	-	-

Supplementary Table 2: Telehealth Usability Questionnaire mapped to the WHO framework for monitoring and evaluating digital health interventions

Telehealth Usability Questionnaire			WHO framework for monitoring and evaluating digital health interventions		
**Domain**	**Question**		**Technology readiness**	**Usability**	**Healthcare impact**
Usefulness	1	I felt this improved my access to healthcare services	-	Acceptance - usefulness	Process improvement - efficiency
	2	I felt this saved (or would save) me time traveling to a hospital or specialist clinic	-	Acceptance - usefulness	Process improvement - efficiency
	3	I felt this provided for my healthcare needs	-	Acceptance - usefulness	Process improvement - quality
Ease of Use	1	It was simple to use the online form	-	Acceptance - ease of use	-
	2	It was easy to learn to use the online form	-	Acceptance - ease of use	-
	3	I was able to navigate the tool quickly when submitting a response.	-	Acceptance - ease of use	-
Quality of Interface	1	The way I interact with the platform (ISLA) hosting the online form is pleasant	Functionality	-	-
	2	I like using the platform (ISLA) hosting the online form	Functionality	-	-
	3	The platform (ISLA) hosting the online form is simple and easy to understand	Functionality	-	-
	4	The platform (ISLA) hosting the online form is able to do everything I would want it to be able to do	Functionality	-	-
Quality of Communication	1	I could easily communicate to the healthcare staff using the platform	-	Acceptance - communication	-
	2	I could easily get a response to the healthcare staff using the platform	-	Acceptance - communication	-
	3	I felt I was able to express myself effectively regarding my wound	-	Acceptance - communication	-
	4	Using the platform, I could communicate with the healthcare staff as well as if we met in person	-	Acceptance - communication	Process improvement - quality
Reliability	1	I think the remote wound reviews provided are the same as in-person wound reviews.	-	-	Process improvement - quality
	2	Whenever I made a mistake using the online form, I could recover easily and quickly	Functionality	-	-
	3	The platform (ISLA) or the online form gave error messages that clearly told me how to fix problems	Functionality	-	-
Satisfaction	1	I feel comfortable communicating with the healthcare staff online	-	Acceptance - satisfaction	-
	2	I found this an acceptable way to receive healthcare services	-	Acceptance - satisfaction	-
	3	I would use the online system again	-	Acceptance - satisfaction	-
	4	I think the online system should be available for others undergoing similar surgery.	-	Acceptance - satisfaction	-
	5	Overall, I am satisfied with the online system	-	Acceptance - satisfaction	-

Supplementary Table 3: Multivariable model of the response rate to the Telehealth Usability Questionnaire, adjusted for case mix

		Response to Telehealth Usability Questionnaire			
		Yes (n=123)	No (n=43)	OR (univariable)	OR (multivariable)
Age	Mean (SD)	48.7 (14.8)	47.5 (18.1)	1.01 (0.98-1.03, p=0.586)	1.01 (0.98-1.04, p=0.604)
Sex	Male	56 (45.5)	25 (58.1)	-	-
	Female	67 (54.5)	18 (41.9)	1.48 (0.73-3.03, p=0.282)	1.69 (0.78-3.76, p=0.187)
Ethnicity	White	121 (98.4)	41 (95.3)	-	-
	BAME	2 (1.6)	2 (4.7)	0.32 (0.04-2.71, p=0.259)	0.29 (0.03-2.73, p=0.251)
Socioeconomic deprivation	Quintile I-II	35 (28.0)	16 (39.0)	-	-
	Quintile III-V	90 (72.0)	25 (61.0)	1.65 (0.78-3.43, p=0.186)	1.56 (0.70-3.42, p=0.272)
Body Mass Index (BMI)	Not obese	79 (64.2)	29 (67.4)	-	-
	Obese	44 (35.8)	14 (32.6)	1.05 (0.50-2.25, p=0.902)	0.92 (0.41-2.09, p=0.841)
Immunosuppression	No	114 (92.7)	40 (93.0)	-	-
	Yes	9 (7.3)	3 (7.0)	0.98 (0.28-4.60, p=0.980)	0.88 (0.23-4.30, p=0.855)
Diabetes Mellitus	No	116 (94.3)	40 (93.0)	-	-
	Yes	7 (5.7)	3 (7.0)	1.33 (0.32-9.08, p=0.723)	1.37 (0.29-10.01, p=0.717)
Operative Urgency	Elective	55 (44.7)	18 (41.9)	-	-
	Emergency	68 (55.3)	25 (58.1)	1.00 (0.49-2.02, p=0.991)	1.05 (0.43-2.57, p=0.921)
Operative approach	Minimally-invasive	74 (60.2)	25 (58.1)	-	-
	Open	49 (39.8)	18 (41.9)	0.94 (0.46-1.95, p=0.868)	0.99 (0.42-2.38, p=0.989)
Operative complexity	Minor/Intermediate	10 (8.1)	3 (7.0)	-	-
	Major	98 (79.7)	35 (81.4)	0.91 (0.20-3.18, p=0.890)	0.97 (0.19-3.76, p=0.971)
	Complex Major	15 (12.2)	5 (11.6)	0.90 (0.16-4.55, p=0.900)	1.09 (0.16-6.60, p=0.925)
Operative contamination	Clean-Contaminated	106 (86.2)	35 (81.4)	-	-
	Contaminated / Dirty	17 (13.8)	8 (18.6)	0.82 (0.33-2.25, p=0.678)	0.86 (0.29-2.68, p=0.787)
30-day surgical-site infection	No	106 (86.2)	35 (81.4)	-	-
	Yes	17 (13.8)	8 (18.6)	0.65 (0.26-1.71, p=0.361)	0.66 (0.25-1.83, p=0.402)
