# Supplementary material for: Evaluation of remote digital postoperative wound monitoring in routine surgical practice
Source: NPJ Digit Med. 2023 May 5;6:85. doi: 10.1038/s41746-023-00824-9 (PMC10161985; doi:10.1038/s41746-023-00824-9)
Supplement: Supplementary file 2 — Reporting Summary [file 41746_2023_824_MOESM2_ESM.pdf]

nature portfolio

Corresponding author(s): DBPR: NPJDIGITALMED-05813R
Last updated by author(s): Mar 14, 2023

Reporting Summary

Nature Portfolio wishes to improve the reproducibility of the work that we publish. This form provides structure for consistency and transparency in reporting. For further information on Nature Portfolio policies, see our Editorial Policies and the Editorial Policy Checklist.

Statistics

For all statistical analyses, confirm that the following items are present in the figure legend, table legend, main text, or Methods section.

n/a	Confirmed	
☐	☒	The exact sample size (*n*) for each experimental group/condition, given as a discrete number and unit of measurement
☐	☒	A statement on whether measurements were taken from distinct samples or whether the same sample was measured repeatedly
☐	☒	The statistical test(s) used AND whether they are one- or two-sided<br>*Only common tests should be described solely by name; describe more complex techniques in the Methods section.*
☐	☒	A description of all covariates tested
☐	☒	A description of any assumptions or corrections, such as tests of normality and adjustment for multiple comparisons
☐	☒	A full description of the statistical parameters including central tendency (e.g. means) or other basic estimates (e.g. regression coefficient) AND variation (e.g. standard deviation) or associated estimates of uncertainty (e.g. confidence intervals)
☐	☒	For null hypothesis testing, the test statistic (e.g. *F*, *t*, *r*) with confidence intervals, effect sizes, degrees of freedom and *P* value noted<br>*Give P values as exact values whenever suitable.*
☒	☐	For Bayesian analysis, information on the choice of priors and Markov chain Monte Carlo settings
☒	☐	For hierarchical and complex designs, identification of the appropriate level for tests and full reporting of outcomes
☐	☒	Estimates of effect sizes (e.g. Cohen's *d*, Pearson's *r*), indicating how they were calculated

Our web collection on statistics for biologists contains articles on many of the points above.

Software and code

Policy information about availability of computer code

Data collection	ISLA Care Ltd
Data analysis	R (v4.1.1)

For manuscripts utilizing custom algorithms or software that are central to the research but not yet described in published literature, software must be made available to editors and reviewers. We strongly encourage code deposition in a community repository (e.g. GitHub). See the Nature Portfolio guidelines for submitting code & software for further information.

Data

Policy information about availability of data

All manuscripts must include a data availability statement. This statement should provide the following information, where applicable:

- Accession codes, unique identifiers, or web links for publicly available datasets
- A description of any restrictions on data availability
- For clinical datasets or third party data, please ensure that the statement adheres to our policy

The datasets generated during and/or analysed during the current study are available from the corresponding author on reasonable request.

Human research participants

Policy information about studies involving human research participants and Sex and Gender in Research.

Reporting on sex and gender	Sex reported with consent
Population characteristics	Sociodemographic and operative data were collected based on clinically relevant risk factors for SSI or potentially disadvantaged populations. These included age, sex, ethnicity (White, or Black, Asian and other minority ethnic groups [BAME]), obesity (body mass index [BMI] ≥ 30 kg/m2), social deprivation (corresponding to the index of multiple deprivation (IMD) quintile 17), diabetes mellitus, immunosuppression (known HIV positive status, corticosteroids, chemotherapy received within 6 weeks, or other immunomodulating drugs), operative approach (open or laparoscopic), operative complexity (minor/intermediate or major / complex major according to the BUPA Schedule of Procedures 18), and CDC surgical wound classification 19 (Clean / Clean-Contaminated, or Contaminated / Dirty).
Recruitment	Adult inpatients (aged ≥16 years) who were consented to undergo abdominal general surgery (at least one surgical incision into the peritoneal cavity or gastrointestinal tract) were screened for eligibility. Key inclusion criteria were smartphone ownership (with internet access) and the capacity to provide informed consent. Patients were excluded based on self-reported visual impairment which would prevent interaction with online resources. Written informed consent for each patient was obtained in line with Good Clinical Practice (GCP) standards. Potential for volunteer bias or bias in those with smartphones (both discussed in paper)
Ethics oversight	West of Scotland Research Ethics Committee (21/WS/0046)

Note that full information on the approval of the study protocol must also be provided in the manuscript.

Field-specific reporting

Please select the one below that is the best fit for your research. If you are not sure, read the appropriate sections before making your selection.

☒ Life sciences ☐ Behavioural & social sciences ☐ Ecological, evolutionary & environmental sciences

For a reference copy of the document with all sections, see nature.com/documents/nr-reporting-summary-flat.pdf

Life sciences study design

All studies must disclose on these points even when the disclosure is negative.

Sample size	N=200. With 104 patients effect sizes of patient agreement in the region of 0.33 were able to be examined at 90% power. Based on previous work 11, a usage rate for the intervention of 65% and a response rate to questionnaires of 80% was anticipated, and so a target sample size of 200 patients was required.
Data exclusions	None
Replication	N/A
Randomization	N/A
Blinding	N/A

Reporting for specific materials, systems and methods

We require information from authors about some types of materials, experimental systems and methods used in many studies. Here, indicate whether each material, system or method listed is relevant to your study. If you are not sure if a list item applies to your research, read the appropriate section before selecting a response.

Materials & experimental systems

n/a	Involved in the study	
☒	☐	Antibodies
☒	☐	Eukaryotic cell lines
☒	☐	Palaeontology and archaeology
☒	☐	Animals and other organisms
☐	☒	Clinical data
☒	☐	Dual use research of concern

Methods

n/a	Involved in the study	
☒	☐	ChIP-seq
☒	☐	Flow cytometry
☒	☐	MRI-based neuroimaging

Clinical data

Policy information about clinical studies

All manuscripts should comply with the ICMJE guidelines for publication of clinical research and a completed CONSORT checklist must be included with all submissions.

Clinical trial registration	NCT05069103
Study protocol	Not published but available on request.
Data collection	The "ImplementatioN of Remote Surgical wOund Assessment During the coviD-19 pandEmic" (INROADE) study was conducted across two tertiary care hospitals in a large health board in the United Kingdom, serving an urban-rural population of over 800,000. between the 1st July 2021 and 30th April 2022
Outcomes	All patients received 30-day postoperative follow-up following a standardised format. Patients who utilised the intervention were asked to complete the Telehealth Usability Questionnaire (TUQ) 20 to evaluate their experience and opinions regarding the intervention. This could be completed online or via telephone
